# Altered eating experience during GLP-1 receptor agonist therapy: a sensory–liking–wanting framework for food preference and nutritional behaviour

**DOI:** 10.3389/fnut.2026.1870484

**Published:** 2026-07-17

**Authors:** Jiayi Du, Zhuo Yang, Yan Chen

**Affiliations:** The Second Hospital of Jilin University, Changchun, China

**Keywords:** dietary intake, eating behaviour, food preference, food reward, GLP-1 receptor agonists, obesity

## Abstract

Eating experience is shaped not only by oral sensory input, but also by hedonic evaluation, interoceptive state, and cue-driven motivation. Glucagon-like peptide-1 receptor agonists (GLP-1RAs), widely used for obesity treatment, offer a clinically relevant model for examining how these processes interact during appetite regulation. Some patients receiving GLP-1RAs report that food tastes different, is less enjoyable, or becomes less tempting, yet these experiences are often grouped together as “taste changes.” This review argues that such reports should be interpreted through a sensory–liking–wanting framework rather than as evidence of a uniform gustatory disorder. We synthesise evidence from peripheral taste signalling, brainstem interoceptive integration, and central food reward pathways. Current evidence does not support a consistent primary impairment of basic gustatory function during GLP-1RA treatment. Instead, altered eating experience appears more consistent with state-dependent food revaluation and reduced reward-driven motivation. However, these responses are heterogeneous and may include limited response, persistent appetite, weight regain, or atypical behavioural phenotypes in some individuals. This framework may help clarify how incretin-based therapies influence food preference, dietary intake, nutritional behaviour, and treatment adherence.

## Graphical abstract

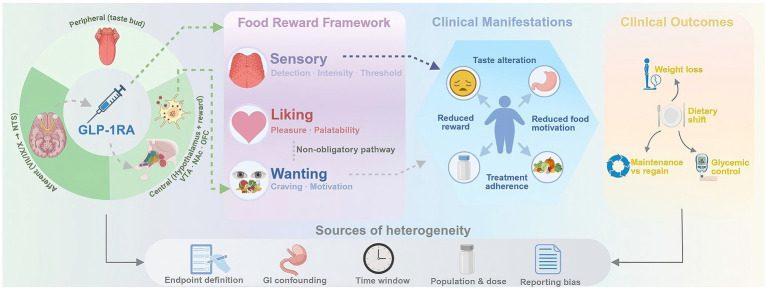

## 1 Introduction

Obesity is a chronic, progressive metabolic disease associated with major cardiometabolic morbidity and growing global prevalence (1, 2). Among anti-obesity medications, glucagon-like peptide-1 receptor agonists (GLP-1RAs) have attracted particular attention. Their effects on satiety, gastric emptying, and energy intake are now well recognised, and large clinical studies have shown meaningful weight-loss benefits in people with overweight and obesity (3, 4). However, with wider clinical use, it has also become clear that the effects of these drugs are not limited to appetite suppression in a narrow sense. Some patients describe noticeable changes in their eating experience during treatment (5). These changes may influence food preference, energy intake, and treatment adherence, and may therefore have important implications for long-term outcomes.

Conventional explanations have largely centred on gastrointestinal–central homeostatic pathways, emphasising the contributions of enhanced satiety and delayed gastric emptying to reduced intake (6). Although this framework may help explain reductions in overall energy intake, it does not fully capture the changes in eating experience perceived by patients. Accumulating evidence indicates that eating behaviour is shaped not only by homeostatic energy demand, but also by the interaction between sensory input and central reward circuitry (7). Early clinical findings suggest that GLP-1RAs may reduce craving for energy-dense foods and alter food preference (3), yet their underlying mechanisms remain insufficiently integrated.

Recent reviews have started to discuss GLP-1RA-related changes in eating behaviour, but most of them mainly focus on outcomes such as body weight, energy intake, craving, or neuropsychiatric adverse events, rather than treating eating experience as a separate issue (8, 9). In addition, patient-reported “taste changes” during GLP-1RA treatment are often discussed in a very general way (10). However, these complaints may not reflect a single phenomenon. They may come from different stages of food-related processing, including basic sensory changes, altered pleasure during eating, or reduced motivation in response to food cues (11). This distinction is important because these changes may involve different mechanisms and may also have different nutritional and clinical implications. So far, however, the literature has not been well organised in a way that clearly separates these different domains when discussing GLP-1RA-related changes in food experience (8).

Against this background, this review examines GLP-1RA-associated changes in eating experience as a multilevel biological phenomenon rather than a uniform “taste disorder.” We use a sensory–liking–wanting framework to organise evidence across peripheral taste signalling, brainstem interoceptive integration, and central reward-related pathways. This framework should be regarded as a conceptual and hypothesis-generating model rather than a validated causal pathway because current human evidence remains heterogeneous in methodology, population, endpoint definition, and follow-up duration. It is also not intended to imply a uniform response to GLP-1RA therapy, as eating-experience changes may vary across individuals and include limited response, persistent appetite, weight regain, or distinct behavioural phenotypes. We further distinguish direct sensory evidence from indirect reward-related and patient-reported evidence in order to clarify the mechanistic boundaries of current knowledge. Through this framework, we aim to provide a more biologically grounded interpretation of altered eating experience during GLP-1RA therapy and to identify key translational and research priorities in obesity treatment.

## 2 Methods

This article was prepared as a structured narrative review of GLP-1 receptor agonists (GLP-1RAs) and altered eating experience during obesity treatment. Literature was searched in PubMed and Web of Science up to March 31, 2026, using combinations of terms including “GLP-1 receptor agonist,” “semaglutide,” “liraglutide,” “tirzepatide,” “obesity,” “taste,” “flavour,” “dysgeusia,” “food preference,” “dietary intake,” “eating behaviour,” “appetite,” “satiety,” “food reward,” “craving,” “liking,” “wanting,” and “interoception.”

Studies were considered relevant if they addressed gustatory sensory function, hedonic food evaluation, food preference, dietary intake, cue-related food motivation, interoceptive modulation, or reward-related neural processing. Human studies were prioritised, while preclinical studies were included when they provided mechanistic evidence relevant to taste signalling, brainstem integration, interoception, or food-reward pathways.

Because the available evidence is heterogeneous in design, population, exposure, and endpoint definition, this review was not designed as a systematic review or meta-analysis. No formal risk-of-bias assessment was performed. Evidence was synthesised narratively and organised using a sensory–liking–wanting framework. Sensory endpoints referred to taste detection, identification, threshold, or intensity; liking endpoints referred to hedonic evaluation, palatability, eating pleasure, or flavour appraisal; and wanting endpoints referred to craving, cue-reactivity, food preoccupation, motivational salience, or reward-driven food seeking.

This framework was used to clarify whether GLP-1RA-associated “taste changes” are better interpreted as alterations in gustatory function, hedonic food evaluation, cue-driven food motivation, or broader nutrition-related changes in food preference and dietary behaviour.

## 3 Biological rationale for a layered model of GLP-1RA-associated changes in eating experience

GLP-1 signalling is implicated not only in classical gut-brain homeostatic regulation of feeding (12), but also potentially at multiple additional levels, including the peripheral taste bud microenvironment (10, 13), gustatory afferent pathways (14–16), and central reward networks (17–21). Overall, these observations support a provisional multilevel framework for considering how GLP-1-related signalling might influence eating experience (11, 22), although evidence is not equally strong across all levels (10).

### 3.1 Taste processing and measurable endpoints

#### 3.1.1 Hierarchical levels of taste-related processing and key concepts

Taste plays a critical role during the early, pre-absorptive phase of food intake (23). To characterise eating behaviour more precisely, taste-related processing can be separated into three relatively distinct but interacting functional levels: sensory, liking, and wanting (11).

Sensory refers to the detection and early encoding of sensory information, including taste, smell, and oral texture, and constitutes the input stage of eating behaviour. This level may directly influence meal initiation and the rate of early intake, while also shaping downstream metabolic responses through oral processing (24). Liking refers to the subjective hedonic evaluation of food flavour. Its neural representation primarily involves regions such as the orbitofrontal cortex (OFC) and is modulated by prior experience, context, and cognitive factors (25). Over short time scales, liking may change with recent eating experience and is closely related to voluntary food intake (26). Wanting, by contrast, reflects the motivational drive and craving for food, and may promote intake even in the absence of a marked increase in pleasure (11). At the population level, wanting is typically expressed as cue-reactivity to food and habitual craving tendency, with inter-individual variation associated with the responsiveness of reward-related brain regions (27).

Importantly, these three levels are related but can still be separated functionally, and they do not always change in the same direction. For example, even when a person is not hungry, external food cues may still trigger wanting and promote food intake, while liking does not necessarily increase at the same time (28). Without separating these levels, different forms of change in eating behaviour may be mistakenly grouped together.

#### 3.1.2 Measurement approaches and confounding factors across endpoint domains

Within this layered endpoint framework, sensory, liking, and wanting correspond to distinct types of measurement. Sensory is primarily assessed using objective gustatory function tests, including Taste Strips and threshold-based methods such as Quick Estimation by Sequential Testing (QUEST) and quick yes–no (qYN) (29, 30). Liking is more closely related to subjective hedonic response and eating satisfaction, and is commonly assessed using food preference and pleasure-rating questionnaires (31, 32). Wanting is typically measured using food craving scales, cue-reactivity paradigms, and assessments of reward sensitivity (33, 34).

It should be noted that all three categories of measurement are vulnerable to common sources of confounding. First, gastrointestinal (GI) symptoms such as nausea and early satiety may substantially distort the assessment of liking and wanting (35). Second, patient-reported “taste change” often reflects the integrated experience of taste, smell, and oral somatosensation, and should therefore be distinguished from pure gustatory function (36). In addition, cognitive expectancy and social desirability bias may also influence subjective ratings (37). These factors should therefore be carefully controlled for, or at minimum considered, in study design and interpretation. Accordingly, studies using psychophysical testing, patient-reported food pleasure, cue-reactivity paradigms, and adverse-event reporting should not be interpreted as equivalent evidence for the same biological construct (Table 1).

**Table 1:** Hierarchical levels of food-related processing and their interpretive relevance.

Level	Core function	Measures/indicators	Interpretation and caveats
Sensory	Early detection and encoding of taste-related oral input	Taste strips; thresholds; intensity/identification tests	Indicates altered taste intensity or quality, but may be confounded by nausea, reflux, xerostomia, or olfactory/flavour disturbance.
Liking	Hedonic evaluation during eating	Hedonic ratings; food pleasure questionnaires; satisfaction ratings	Indicates reduced food enjoyment or satisfaction, but is influenced by satiety and gastrointestinal discomfort.
Wanting	Motivational salience and cue-triggered desire to eat	Craving scales; cue-reactivity tasks; food-cue imaging	Indicates reduced food temptation or interest, but may be inferred indirectly or reflect aversive suppression.

### 3.2 Peripheral GLP-1 signalling in taste pathways: cellular plausibility and translational limits

Preclinical studies indicate that GLP-1 is produced in distinct subpopulations of mammalian taste bud cells (TBCs) (13), including TAS1R3-positive and gustducin-positive type II cells, as well as a subset of serotonin-containing type III cells (38). This cellular distribution suggests that GLP-1 is embedded within the local taste bud microenvironment and may participate in gustatory processing at an early pre-central stage rather than acting solely as a distal metabolic signal.

GLP-1 acts on neighbouring GLP-1R-positive afferent nerve terminals within the taste bud, thereby forming a local regulatory circuit that influences the early encoding of taste signals (13). As a G protein-coupled receptor (GPCR), GLP-1 receptor (GLP-1R) activation may further regulate downstream signalling pathways, including protein kinase A (PKA) and exchange protein directly activated by cAMP (EPAC), which may influence afferent excitability, signal gain, or synaptic-like communication between taste receptor cells and gustatory nerve endings (39). From a mechanistic perspective, such effects would be more consistent with modulation of sensory weighting or encoding efficiency than with the generation of an entirely new sensory quality.

Functional studies suggest that the GLP-1/GLP-1R axis can alter the firing properties of specific gustatory nerve fibres and modulate early afferent taste signalling, particularly in preclinical models (13, 40). These findings support the biological plausibility of a peripheral sensory contribution to GLP-1RA-associated changes in eating experience, but they do not establish that local taste-pathway modulation is sufficient to explain stable or consciously perceived alterations in flavour, food pleasure, or food-directed motivation in humans. Thus, peripheral taste-pathway modulation should be interpreted as one biologically plausible component of altered eating experience, but not as a sufficient explanation for the full human phenotype.

### 3.3 Localisation of endogenous GLP-1 and GLP-1 receptors in the rodent and human brain

Endogenous GLP-1 is not exclusively derived from peripheral intestinal L-cells; it also constitutes a relatively independent neuropeptidergic regulatory system within the central nervous system. Rodent studies have shown that brain-derived GLP-1 is primarily produced by neurons in the lower brainstem that express preproglucagon (PPG), with a particular enrichment in the nucleus of the solitary tract (NTS) and adjacent medullary regions. Selective ablation of NTS PPG neurons significantly reduces active GLP-1 levels in both the brain and spinal cord, suggesting that these neurons represent a major source of endogenous central GLP-1 (41). Correspondingly, GLP-1Rs are widely, yet region-specifically, distributed throughout the rat brain, including the olfactory bulb, cerebral cortex, hippocampus, lateral septum, amygdala, nucleus accumbens, multiple hypothalamic nuclei (such as the paraventricular nucleus, supraoptic nucleus, arcuate nucleus, and dorsomedial nucleus), ventral tegmental area, substantia nigra, raphe nuclei, parabrachial nucleus, locus coeruleus, nucleus tractus solitarius, area postrema, and dorsal motor nucleus of the vagus nerve. This distribution pattern suggests that GLP-1Rs may be involved in the integration of visceral sensory signals, regulation of energy homeostasis, reward-related behaviours, and autonomic nervous system control (42).

In human brain tissue, GLP-1Rs have similarly been confirmed in neurons within the hypothalamus, medulla oblongata, and parietal cortex. More specifically, GLP-1R expression has been identified in several hypothalamic and basal forebrain regions, including the paraventricular nucleus, supraoptic nucleus, infundibular nucleus/arcuate nucleus, lateral hypothalamic area, and nuclei of the basal forebrain, where they exhibit relatively well-defined regional expression patterns (43, 44). Notably, GLP-1R mRNA expression is reduced in the paraventricular nucleus and infundibular nucleus of the hypothalamus in patients with type 2 diabetes mellitus (T2DM) (45). In individuals with elevated body mass index (BMI), GLP-1R immunoreactivity in the lateral hypothalamic area is decreased and negatively correlated with BMI. Moreover, GLP-1R immunoreactivity is predominantly co-localised with the anorexigenic neuropeptide NUCB2/nesfatin-1, rather than with the astrocyte marker glial fibrillary acidic protein (GFAP) (44). Collectively, these findings indicate that the central GLP-1/GLP-1R system may function as more than a passive relay of peripheral metabolic information. Instead, it appears to contribute to the regulation of eating experiences, energy homeostasis, and obesity- and diabetes-related dysregulation of feeding through integrated brainstem–hypothalamic–cortical/reward circuitry.

### 3.4 Brainstem integration of gustatory and interoceptive signals

#### 3.4.1 Anatomy of gustatory afferent pathways and signal convergence

Following transduction by taste receptor cells, peripheral gustatory information is conveyed to the brainstem via afferent fibres associated with the facial nerve (VII, chorda tympani), glossopharyngeal nerve (IX), and vagus nerve (X) (15) and undergoes primary integration in the NTS (46). Neuroimaging studies further show that taste stimulation can elicit activity changes within the NTS region of the brainstem, supporting its role as an early integrative node for gustatory information (16). Accordingly, the NTS provides a key anatomical and functional substrate for understanding how metabolic signals may influence taste input at the afferent stage. This convergence provides an anatomical substrate through which the meaning of oral sensory input may be modified by current physiological state before higher-order valuation occurs.

#### 3.4.2 NTS as an integrative node for taste and interoceptive state

In addition to integrating gustatory information, the NTS receives chemical signals from the oropharynx and gastrointestinal tract, thereby serving as a hub for the early coupling of oral cues, visceral feedback, and feeding output (47). Through its projection neurons, the NTS relays integrated information to the hypothalamus and other forebrain regions, thereby coordinating feeding behaviour and associated physiological responses (48). The NTS also transmits taste-related information to forebrain regions including the hypothalamus, and can drive feeding under hypoglycaemic conditions (49). In addition, NTS neurons projecting to multiple forebrain regions contribute to both physiological and emotional regulation (50). In summary, these studies support the NTS as an early integrative node for gustatory and interoceptive signalling (51, 52).

#### 3.4.3 Brainstem GLP-1R expression and functional boundaries

At the brainstem level, GLP-1 receptors (GLP-1Rs) are expressed in multiple neuronal populations within the NTS, including PPG neurons and GABAergic neurons (22). Their activation can increase the excitability of specific neuronal populations, thereby altering neural responses to food-related cues.

In addition, endogenous GLP-1 neurons in the NTS project to the ventral tegmental area (VTA) and nucleus accumbens (NAc), pathways involved in food reward processing and the regulation of eating motivation and hedonic experience (18). Beyond serving as a relay to mesolimbic reward circuits, the NTS itself appears to actively participate in the regulation of hedonic feeding. Extending this anatomical link, pharmacological evidence shows that activation of GLP-1Rs within the VTA, NAc core, and NAc shell reduces food intake, particularly intake of highly palatable foods (18). Importantly, direct activation of GLP-1Rs within the NTS also suppresses food reward behaviour, reduces operant responding for sucrose, abolishes conditioned place preference for palatable food, and preferentially decreases palatable food intake when animals are given a choice between palatable food and standard chow (53). These effects were not accompanied by reduced locomotor activity or nausea-like behaviour, suggesting that NTS GLP-1R signalling can modulate motivational and reward-related components of feeding rather than merely producing nonspecific malaise (53).

Together, these findings indicate that the NTS is not merely a passive relay of visceral information, but can actively influence both homeostatic and hedonic components of feeding. At the same time, NTS–vagal pathways also contribute to clinically relevant GLP-1RA-associated effects, including reduced intake, enhanced satiety, and nausea (54). Thus, the brainstem—particularly the NTS—is best understood here as an active integrative hub through which gustatory input may be reweighted by interoceptive and visceral state signals during GLP-1RA treatment, rather than as direct evidence of taste-specific dysfunction in humans.

### 3.5 Central mechanisms of food revaluation: homeostatic and reward-related pathways

#### 3.5.1 The NTS–hypothalamic homeostatic pathway and suppression of intake

In addition to peripheral sources, an independent GLP-1 signalling system exists within the central nervous system, centred on PPG-expressing neurons in the NTS (22). This system is, to some extent, functionally distinct from peripheral GLP-1 and contributes to the central regulation of feeding suppression. As a key centre of energy homeostasis, the hypothalamus integrates multiple metabolic and interoceptive signals to regulate the initiation and inhibition of feeding (55). Under hypoglycaemic conditions, catecholaminergic neurons in the NTS can regulate agouti-related peptide (AgRP)/pro-opiomelanocortin (POMC) neuronal activity in the arcuate nucleus (ARC) via ascending projections, thereby driving feeding behaviour (56). This homeostatic pathway primarily influences outputs such as total energy intake and satiety and does not necessarily imply alteration in sensory input per se. Interpretation of GLP-1RA-associated changes in eating experience requires separation of homeostatic suppression of intake from alterations in the subjective experience of food. Homeostatic inhibition of feeding is highly relevant to reduced energy intake, but it should not be assumed to explain patient-reported changes in food pleasure, flavour experience, or cue-triggered food motivation.

#### 3.5.2 GLP-1 modulation within reward networks and downregulation of motivation

The reward network can be conceptualised as a distributed circuit involving the ACC, OFC, ventral striatum, ventral pallidum, and midbrain dopaminergic systems, with additional modulation by structures such as the amygdala and hippocampus (54, 55).

Direct evidence comes from two main sources. First, preclinical studies indicate that GLP-1R activation can reduce excitatory synaptic input onto VTA dopamine neurons and suppress intake of highly rewarding foods (56–58). Second, human studies have reported reduced neural responses to palatable food cues during GLP-1RA treatment in regions including the insula and OFC (59–61).

These findings are more compatible with altered food valuation and reduced cue-driven motivation than with a primary change in basic gustatory processing. However, the human evidence remains largely indirect, because neural cue reactivity and self-reported craving do not isolate wanting as a completely separable construct.

#### 3.5.3 Revaluation of reward under state signals

However, reward-related downregulation should be distinguished from nausea- or discomfort-related suppression of eating. Eating experience depends not only on peripheral sensory input but also on interoceptive and emotional state signals. Neuroimaging studies have shown that hunger enhances responses to food stimuli in the amygdala and ventromedial prefrontal cortex (vmPFC), thereby increasing subjective food value and craving (62). By contrast, stress reduces sensitivity to rewarding outcomes in the striatum and OFC, manifesting as impaired value evaluation (63).

GLP-1RAs may alter interoceptive state through multiple mechanisms. For example, they may induce nausea through brainstem pathways (64) and enhance satiety through delayed gastric emptying (65). These state signals may alter subjective hedonic evaluation (liking) and motivational drive (wanting) without necessarily affecting basic gustatory discrimination.

**Figure 1:**
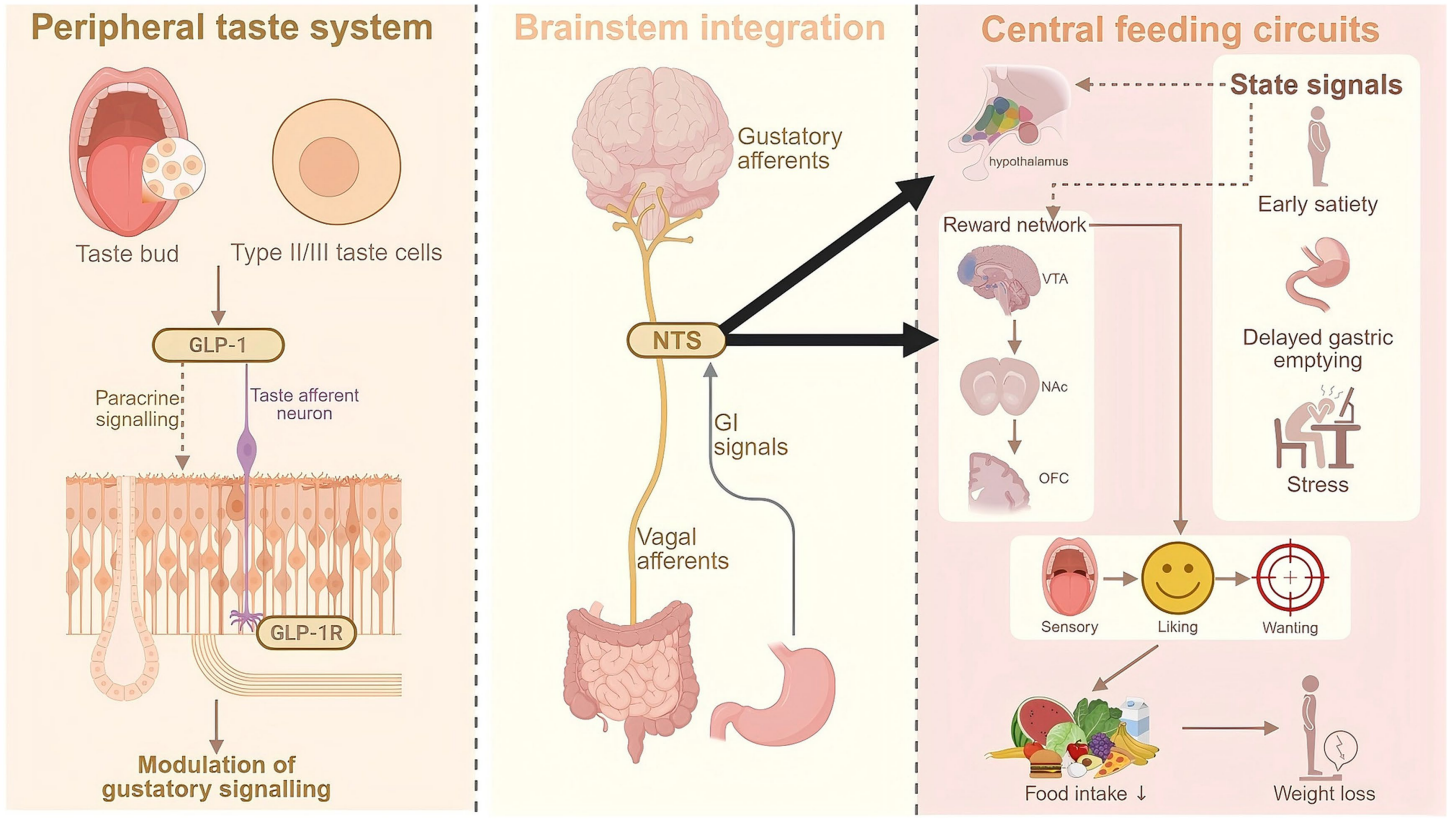
Multilevel mechanisms linking GLP-1 signalling, taste processing and feeding behaviour. GLP-1 signalling modulates food intake through coordinated actions across peripheral, brainstem and central neural circuits. In the peripheral taste system, GLP-1 produced by Type II/III taste cells acts in a paracrine manner on GLP-1 receptors (GLP-1R) located on taste afferent nerve terminals, thereby modulating gustatory signalling. Taste information is transmitted via gustatory afferents to the nucleus of the solitary tract (NTS), where it is integrated with gastrointestinal (GI) signals conveyed by vagal afferents. From the NTS, integrated signals are relayed to central feeding circuits, including hypothalamic homeostatic centres and the mesolimbic reward network (VTA–NAc–OFC). These circuits interact with physiological state signals such as early satiety, delayed gastric emptying and stress. Through these integrated pathways, GLP-1 signalling influences sensory perception, hedonic evaluation (*liking*) and motivational drive (*wanting*), ultimately reducing food intake and contributing to weight loss.

Nausea-related feeding suppression is not equivalent to reduced motivation mediated by central reward pathways. The former is more likely to reflect a non-specific aversive response or conditioned avoidance, whereas the latter involves altered incentive salience and subjective value of food cues, and may therefore be more selective and potentially more persistent. Failure to distinguish between these mechanisms may result in oversimplified or misleading interpretations of GLP-1RA action (Figure 1).

## 4 Evidence framework and phenotypic heterogeneity of GLP-1RA-associated taste-related changes

In real-world obesity treatment, some patients receiving GLP-1RAs report changes in eating experience, such as “food tastes different,” “food is no longer appealing,” or the emergence of unpleasant taste sensations (10). However, the current evidence base is characterised by substantial heterogeneity in endpoint definition, measurement approach, and mechanistic attribution. On the one hand, studies have used inconsistent terminology and ascertainment strategies for these outcomes, ranging from dysgeusia or taste disorder reported as adverse events (AEs) (66) to broader concepts such as food taste change or patient-reported alterations in flavour evaluation (67). On the other hand, taste-related complaints frequently co-occur with gastrointestinal symptoms such as nausea and early satiety (35, 68), making it difficult to distinguish between objective gustatory dysfunction (sensory) and reward revaluation driven by state-related signals (liking/wanting) at the clinical level. Accordingly, much of the heterogeneity in the existing literature may reflect differences in endpoint level, ascertainment strategy, and control of confounding, rather than the absence of a biological effect.

### 4.1 Trial evidence: recording and limitations of taste-related outcomes in RCTs and PROs

In randomised controlled trials (RCTs), primary outcomes are typically focused on body weight and metabolic endpoints, whereas eating-experience-related outcomes are rarely included as prespecified measures and are more often captured only sporadically as adverse events (AEs) (69). By contrast, gastrointestinal events such as nausea and vomiting are generally recorded more systematically and are commonly treated as key tolerability endpoints (68).

Patient-reported outcomes (PROs) may provide a more sensitive window into subjective changes in eating experience, particularly with respect to hedonic evaluation (liking) and food preference (70). However, the tools currently used for these assessments lack standardisation, and considerable variation exists across studies in endpoint definition, assessment window, and measurement method.

In addition, follow-up duration is short in most RCTs (71, 72), limiting assessment of the persistence, reversibility, and temporal dynamics of altered eating experience. Taken together, current RCT and PRO data suggest that GLP-1RA treatment is associated with changes in eating experience, but remain insufficient to determine the specific functional level involved (sensory, liking, or wanting) or the long-term clinical significance of these changes.

### 4.2 Real-world evidence: signal strength and heterogeneity of evidence

Real-world evidence is derived primarily from spontaneous reporting systems and cohort studies. Spontaneous reporting databases typically use indices such as reporting odds ratios (RORs), proportional reporting ratios (PRRs), or information components (ICs) to estimate the relative reporting tendency of adverse events; these measures reflect reporting intensity rather than true incidence (73). Such data are also highly susceptible to under-reporting, selective reporting, and media attention, and are therefore better suited to signal detection than to risk quantification (74).

By contrast, cohort studies can provide estimates of incidence and relative risk through better-defined exposed populations and follow-up windows (75). However, in routine clinical practice, changes in eating experience are often reported subjectively (76), and may be difficult to distinguish from olfactory changes, xerostomia, or gastrointestinal discomfort (68, 77), thereby compromising the precision of endpoint definition.

Overall, the heterogeneity of real-world evidence appears to arise from three main sources: spontaneous reporting data are highly sensitive to media attention and prescribing volume, such that signal strength does not equate to true risk (74); patient-reported changes in eating experience often combine olfactory, oral somatosensory, and gastrointestinal components, making endpoint harmonisation difficult across studies (76); and, in the context of obesity treatment, differences in dose, titration speed, co-occurring nausea, and baseline oral health may further amplify inter-individual phenotypic variation. It is therefore unsurprising that studies differ in reported incidence, direction of effect, and mechanistic interpretation.

### 4.3 Risk-related factors: exposure characteristics, co-occurring symptoms, and baseline susceptibility

Within the current evidence base, the occurrence and phenotype of GLP-1RA-associated changes in eating experience can be interpreted as being shaped by exposure characteristics, co-occurring symptoms, and individual susceptibility. With regard to exposure, more rapid dose escalation or greater overall exposure may increase gastrointestinal symptom burden (72), thereby promoting avoidance of specific foods (78) and increasing the likelihood of related complaints. In terms of co-occurring symptoms, altered eating experience frequently coexists with gastro-oesophageal reflux, early satiety, and reduced appetite (79), suggesting that in a substantial proportion of patients these phenomena may reflect state-dependent responses rather than isolated sensory abnormalities. At the level of individual susceptibility, pre-existing reflux, oral dryness, or olfactory/gustatory dysfunction may increase the likelihood of such complaints (77, 79, 80). Although these factors do not directly determine mechanistic pathways, they may influence both symptom perception and reporting.

Mechanistic studies to date suggest that GLP-1 signalling is more likely to modulate taste-related and eating-experience-related processing than to cause permanent sensory injury (13). This interpretation is consistent with clinical observations that some symptoms diminish or resolve over time, suggesting that in a substantial proportion of patients they may reflect a reversible adaptive process. Thus, much of the observed heterogeneity in reported “taste changes” may reflect differences in endpoint definition, symptom context, and measurement, rather than consistent evidence of a single underlying mechanism.

## 5 Clinical and translational evidence: differential evidence across eating-experience-related endpoints during GLP-1RA treatment

Across the current literature, GLP-1RA-associated eating-experience outcomes differ substantially in both definition and level of measurement. Some studies assess objective gustatory function (10), whereas others examine subjective food pleasure (5). Still others evaluate food reward motivation or craving (81). In the present Review, these findings are mapped onto the sensory–liking–wanting framework as an interpretive strategy to improve comparability across heterogeneous endpoint domains (11). This mapping should not be taken to mean that the original studies themselves were designed around this framework or that the boundaries between these domains are always sharp (9).

### 5.1 Sensory: evidence and limitations of objective gustatory endpoints

Studies directly assessing the effects of GLP-1RAs on objective gustatory function (sensory) remain limited. A small number of translational and clinical studies have used psychophysical methods, including taste thresholds, taste-quality identification, and composite taste scores, and suggest that some individuals may show measurable changes in gustatory function during treatment (10, 82). However, these findings are notably inconsistent across studies.

Sensory endpoints are themselves subject to several important limitations. First, psychophysical taste testing is highly sensitive to physiological state and may be influenced by nausea, gastro-oesophageal reflux, oral dryness, and changes in olfactory function (83), all of which are relatively common during early GLP-1RA treatment. Second, most available studies have relatively short follow-up (82, 84), making it difficult to distinguish transient state-dependent fluctuation from sustained sensory change. In addition, some studies do not clearly distinguish between taste and flavour, meaning that alterations in olfaction or oral somatosensation may be misattributed to gustatory dysfunction (10).

Accordingly, the current evidence is insufficient to conclude that GLP-1RAs produce a consistent and stable alteration in basic gustatory sensory function. The sensory findings reviewed here are heterogeneous and vulnerable to multiple confounders. One possible interpretation is that peripheral sensory modulation, if present, represents only one component of a broader process rather than a uniformly demonstrated primary mechanism.

### 5.2 Liking: evidence for subjective taste and eating-experience endpoints

Compared with sensory endpoints, evidence relating to the effects of GLP-1RAs on subjective hedonic evaluation (liking) is more abundant and derives mainly from patient-reported outcomes (PROs) and eating-experience-related questionnaire studies. Existing RCTs and prospective studies suggest that some participants report reduced eating pleasure or altered flavour evaluation during treatment, often alongside reduced appetite and early satiety (5).

Variation across studies appears to arise largely from differences in endpoint definition and measurement strategy. On the one hand, some studies capture dysgeusia or taste disorder as adverse events (AEs) (66), which may reflect abnormal sensation or treatment-related discomfort. On the other hand, PRO-based studies tend to assess food taste change, eating pleasure, or meal satisfaction, which are conceptually closer to the liking domain (67). Differences in instruments, observation windows, and study populations may further contribute to inconsistency. Thus, the same underlying phenomenon may be described as either “taste abnormality” or “reduced pleasure,” depending on the level and method of assessment, and these interpretations are not necessarily contradictory.

Collectively, the available studies support the view that GLP-1RA treatment can be accompanied by changes in eating pleasure or flavour evaluation in at least some individuals. A reasonable interpretation is that these changes often reflect revaluation of food under altered interoceptive conditions, such as satiety or gastrointestinal discomfort, rather than direct evidence of altered gustatory input. However, this remains an interpretive inference because most studies do not experimentally separate these pathways.

### 5.3 Wanting: evidence for reward motivation and craving-related endpoints

Changes in liking do not necessarily imply changes in wanting (11). Therefore, when evaluating the effects of GLP-1RAs on eating behaviour, it is necessary to consider whether motivational aspects of wanting are also altered. Across randomised and prospective studies, the overall pattern suggests that GLP-1RA treatment reduces craving and the desire to consume energy-dense foods in at least a proportion of participants, alongside reductions in overall appetite and energy intake (17, 81). Beyond self-report measures, some studies have used cue-reactivity paradigms and neuroimaging methods and further observed attenuated reward responses to high-calorie food cues (60).

Wanting-related endpoints are, however, also heterogeneous. First, different studies do not measure the same construct of wanting: some emphasise immediate craving or desire (85), whereas others focus on food preoccupation (86), limiting direct comparability. Second, differences in drug type, dose, and population characteristics may further contribute to inconsistency in effect size and presentation. Even so, several studies suggest a tendency toward reduced craving or cue-related food motivation during GLP-1RA treatment, although the magnitude, durability, and specificity of these effects vary across measures, populations, and study designs. This pattern is also more closely aligned with evidence for central reward-system modulation, including reduced neural responsiveness to food cues and attenuation of activity in reward-related brain regions. These findings are broadly compatible with the possibility that GLP-1 signalling may attenuate cue-driven incentive salience, but they do not yet establish a single dopaminergic mechanism as the definitive explanation for the observed behavioural changes.

Even with these limitations, wanting-related measures show a relatively recurring pattern of reduced craving or reduced responsiveness to highly palatable food cues across several studies. These findings are compatible with a reward-related mechanism, potentially involving reduced cue-driven incentive salience. However, the link from altered neural cue responsivity to wanting as a specific psychological construct remains partly inferential. Therefore, a cautious summary is that current evidence appears more compatible with an effect on reward and motivational processing than with a consistent direct alteration in basic taste perception, while acknowledging that these pathways have not been definitively separated in humans.

## 6 A proposed framework for interpreting GLP-1RA-associated changes in eating experience

Current evidence suggests that changes in eating experience during GLP-1RA treatment are unlikely to come from only one mechanism or one single level of processing. Instead, the existing literature seems to support a more complex picture, in which peripheral sensory modulation, changes related to interoceptive state, and altered food-directed motivation may all play a role, although their relative importance may differ across patients and across different stages of treatment. In this review, this framework is mainly used as a way to organise findings from preclinical, translational, and clinical studies, rather than as proof of a fully established causal pathway in humans. Causal relationships among GLP-1RA exposure, peripheral taste-pathway modulation, central reward-circuit activity, altered liking, reduced wanting, and long-term dietary behaviour remain incompletely established, and alternative explanations such as nausea-related aversion, delayed gastric emptying, altered satiety, expectancy effects, dietary compensation, medication adherence, and psychological or behavioural moderators should also be considered. From this perspective, reported “taste changes” may be better understood as reflecting different parts of a broader eating-experience continuum, ranging from sensory input to hedonic evaluation and motivational drive.

### 6.1 A possible peripheral contribution to altered eating experience

Basic studies indicate that GLP-1-related signalling components are present within taste buds and may participate in the local modulation of gustatory information, particularly in pathways related to sweet taste and energy intake (87). These observations suggest that the peripheral taste system may represent one potential entry point for GLP-1RA effects.

However, taste perception does not operate as an isolated sensory input; rather, it reflects a highly integrated process involving olfaction, oral somatosensation, and interoceptive state (88). Accordingly, even if mild changes in peripheral taste sensitivity do occur, their expression at the level of subjective experience or behaviour may be unstable. In addition, psychophysical taste testing is highly sensitive to the physiological state of the participant and may be influenced by nausea, early satiety, and changes in the oral environment (83). This makes peripheral gustatory modulation difficult to capture consistently in clinical studies.

Viewed together, peripheral taste pathways may contribute to GLP-1RA-associated effects, but they are unlikely to account on their own for the broader and more systematic changes observed in eating experience and behaviour. Rather, they are more plausibly understood as one component within a wider multi-mechanistic network.

### 6.2 Central integration and value adjustment: coordinated regulation by homeostatic and reward pathways

Compared with peripheral mechanisms, central integrative pathways provide a more comprehensive explanation for the systemic effects of GLP-1RAs on eating experience and behaviour. GLP-1-related signals may be integrated through the brainstem–hypothalamic axis with interoceptive input arising from gastrointestinal and metabolic states, thereby modulating the homeostatic drive to eat (89).

Within this framework, interaction between homeostatic and reward systems provides a key model for understanding altered eating experience (11, 90). Homeostatic signals provide the background state within which reward processing occurs, whereas reward systems determine the subjective value of food and the strength of motivational drive.

Accordingly, one plausible interpretation is that GLP-1RAs may reduce the overall subjective appeal of food through satiety-related interoceptive signals and attenuation of hunger drive, thereby manifesting as changes in liking (5). At the same time, GLP-1RAs may reduce central responses to cues associated with energy-dense foods, thereby lowering cue-triggered motivational drive (wanting) (5, 60).

Importantly, liking and wanting do not necessarily follow the same temporal pattern and may differ across individuals. Liking is more dependent on immediate context and momentary experience, whereas wanting may be more readily expressed as a broader downward shift in cue-driven responsivity. Thus, discrepancies across studies in these two domains may reflect differences in measurement level and observation window rather than fundamentally different biological processes.

### 6.3 State dependence and learning mechanisms: avoidance and remodelling of motivation

In addition to peripheral and central pathways, state-dependent factors and learning processes are also likely to contribute to changes in eating experience. Gastrointestinal discomfort and early satiety may directly alter the immediate subjective experience of eating, such that foods are perceived as less desirable or more difficult to consume (81). If specific foods are repeatedly paired with discomfort, individuals may develop negative associative links through conditioned learning, thereby reducing the subjective attractiveness of those foods. This process more likely reflects revaluation of eating outcomes than a direct change in taste sensitivity (11).

Such learning effects may extend beyond the act of eating itself to the cue stage. Once food-related cues acquire negative predictive value, they may trigger avoidance responses and lead to an earlier decline in motivational drive (wanting) (91). Importantly, this process is typically state-dependent and reversible, and should not be equated with pathological gustatory damage. Available evidence suggests that GLP-1-related signalling is more likely to participate in dynamic modulation than to induce irreversible sensory dysfunction (13).

### 6.4 A layered and stage-sensitive working model

Taken together, GLP-1RA-associated changes in eating experience can be conceptualised as a layered and stage-dependent mediating pathway. Drug effects are unlikely to depend on a single entry point; rather, within this framework, observed clinical phenomena may reflect contributions from peripheral modulation, central integration, and state-dependent processes to varying degrees across treatment stages, influencing multiple nodes including sensory, liking, and wanting, and ultimately shaping food intake and body-weight outcomes (11). Accordingly, patient-reported complaints such as “food tastes different,” “food is less enjoyable,” or “I no longer want to eat” may reflect different points along this mediating chain: some may correspond to subjective value reappraisal under altered interoceptive states (5), whereas others may reflect reduced cue responsivity or motivational suppression arising from avoidance learning (5, 60). Thus, superficially similar clinical complaints may map onto distinct mediating processes, underscoring the importance of distinguishing between sensory, liking, and wanting in both research and clinical assessment.

This mediating pathway may also have a temporal dimension. Early in treatment, state signals such as gastrointestinal discomfort and enhanced satiety may predominate, manifesting as short-term reductions in liking or food avoidance. As adaptation occurs, these effects may diminish, whereas reduced motivational drive towards highly rewarding foods (wanting) may persist in at least some individuals (60, 61). Therefore, differences across studies in effect size and phenotypic presentation may arise from variation in observation window, endpoint level, and control of state-related confounding, rather than from genuine inconsistency in the underlying mechanism.

**Figure 2:**
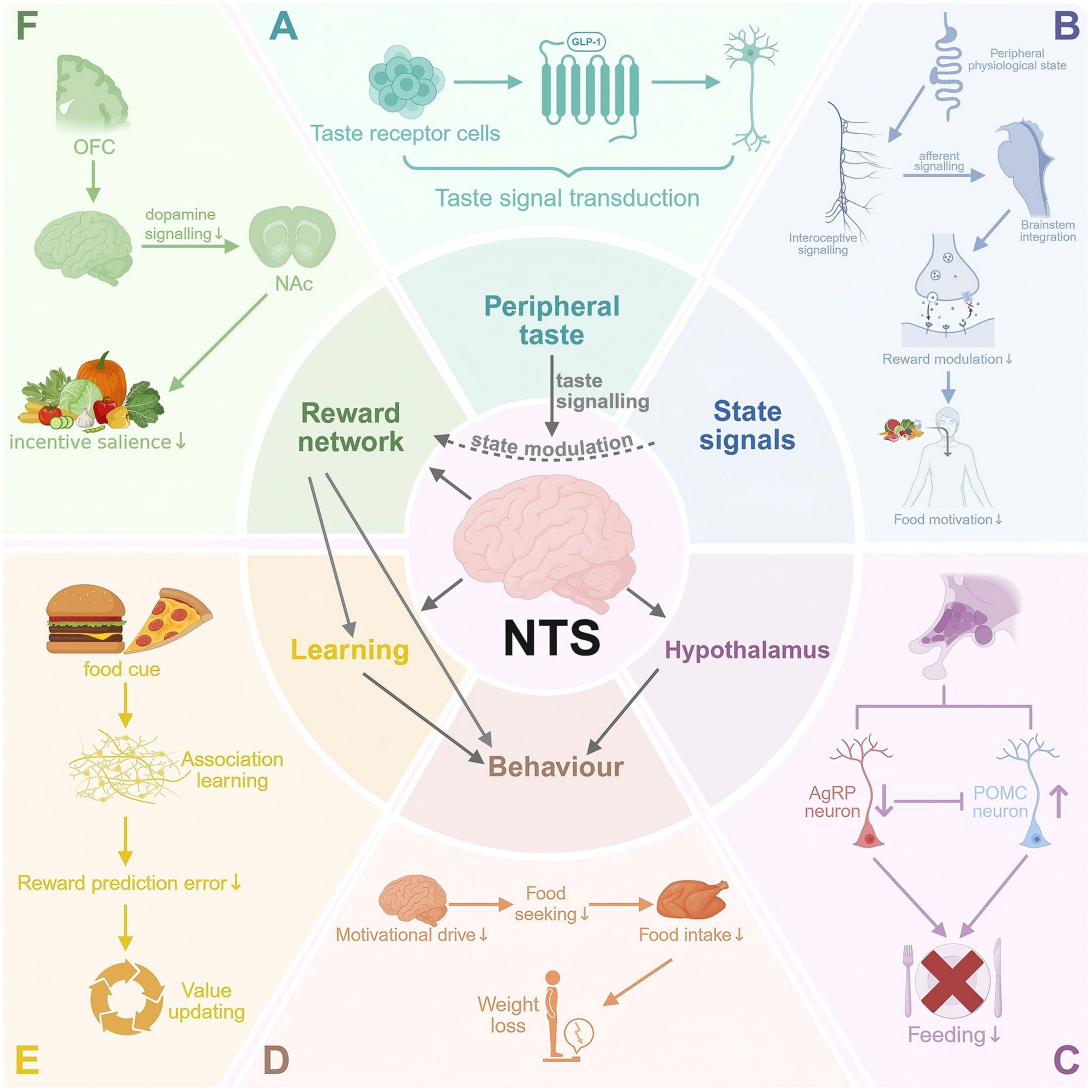
Integrated neural circuits through which GLP-1 signalling regulates feeding behaviour. **(A)** In the peripheral taste system, GLP-1 released from taste receptor cells activates GLP-1 receptors and modulates taste signal transduction, transmitting sensory information to the brainstem. **(B)** Physiological state signals, including gastric distension, gut hormone signalling and other interoceptive inputs, reach the brainstem via afferent pathways and influence central reward processing. **(C)** In the hypothalamus, GLP-1 signalling regulates homeostatic feeding circuits by inhibiting AgRP neurons and activating POMC neurons in the arcuate nucleus, promoting satiety and suppressing feeding. **(D)** Integration of these signals ultimately reduces motivational drive, food seeking and food intake, contributing to body weight loss. **(E)** The learning system updates the value of food-related cues through associative learning and reward prediction error signalling, weakening cue-driven food motivation. **(F)** Within the reward network, GLP-1 signalling modulates the orbitofrontal cortex (OFC)–nucleus accumbens (NAc) circuit and attenuates dopaminergic signalling, thereby reducing the incentive salience of food cues. These peripheral and central pathways converge in the nucleus of the solitary tract (NTS), which serves as a key integration hub for feeding regulation.

The integrated model does not exclude a contribution from peripheral sensory effects; rather, it provides one possible framework in which such effects may be embedded within a broader multi-level process that could, in some individuals or contexts, be shaped more strongly by central valuation and motivational mechanisms (Figure 2).

## 7 Translational and phenotype-informed implications of altered eating experience during GLP-1RA treatment

GLP-1RA-associated changes in eating experience are clinically heterogeneous, and their significance is likely to vary according to individual susceptibility and stage of treatment. Patient-reported complaints often span multiple domains, including sensory perception, eating pleasure, and interest in food. If these experiences are not differentiated, they may be broadly labelled as “taste abnormalities,” potentially leading to imprecision in both mechanistic interpretation and clinical decision-making. The following points are intended as translational implications of the current framework, rather than as formal guideline recommendations.

### 7.1 Interpreting patient-reported “taste change” across sensory, hedonic, and motivational levels

Patient-reported “taste changes” during GLP-1RA treatment likely encompass multiple levels of food-related processing rather than a single uniform abnormality (11). In some cases, complaints centred on altered intensity or impaired identification of specific taste qualities may be more compatible with changes at the sensory level (10). By contrast, reports that food is “less enjoyable” during eating are conceptually closer to altered hedonic evaluation (liking), whereas statements such as “I no longer want food” or “food is less tempting even when I see it” are more consistent with reduced motivational salience (wanting). This distinction is important because superficially similar complaints may arise from different mechanisms and should not be interpreted as equivalent evidence of gustatory dysfunction (76).

### 7.2 Translational implications for endpoint interpretation and functional relevance

From a translational perspective, the major challenge is not simply whether altered eating experience occurs, but how it should be interpreted across endpoint levels (11). Subjective reports of “taste change” do not necessarily indicate primary impairment of gustatory function, particularly when they occur alongside nausea, early satiety, reflux, oral dryness, or broader alterations in flavour perception (76). Instead, many such reports may be more consistent with state-dependent revaluation of food or reduced cue-driven motivation (5). Their practical relevance depends less on the complaint itself than on whether they are associated with altered food choice, reduced overall intake, or treatment adherence (92). Accordingly, distinguishing sensory, hedonic, and motivational phenotypes may improve interpretation of both patient-reported outcomes and mechanistic studies (11).

### 7.3 Temporal dynamics and adaptation

The interpretation of altered eating experience during GLP-1RA treatment is also likely to depend on time course (70). Early in treatment, gastrointestinal discomfort, enhanced satiety, and other interoceptive state signals may predominate and contribute to transient reductions in food appeal or avoidance of specific foods (35). Over time, some of these early effects may diminish with adaptation, whereas reduced responsiveness to highly rewarding food cues may persist in a subset of individuals (60). This temporal dimension is relevant because early aversive suppression and later motivational downregulation are unlikely to be mechanistically identical, even when both are expressed clinically as reduced interest in food. Longitudinal assessment frameworks that distinguish these phases may therefore be important for understanding individual variability in treatment response (70).

### 7.4 Clinical interpretation of divergent response phenotypes

From a clinical perspective, patient-reported eating-experience changes during GLP-1RA therapy should be interpreted in relation to the dominant response phenotype rather than assumed to reflect a uniform treatment effect. In typical responders, reduced appetite, lower food-cue reactivity, decreased preference for energy-dense foods, and weight loss may reflect coordinated changes in satiety, interoceptive state, and food valuation. In contrast, limited weight loss or persistent appetite should prompt assessment of medication exposure, dose escalation, gastrointestinal tolerability, adherence, treatment interruption, concomitant medications, dietary compensation, and behavioural drivers of eating, consistent with evidence that early response may help identify patients more likely to achieve clinically meaningful longer-term weight loss (93). Reports of persistent craving, emotional eating, binge-like eating, or continued intake of highly palatable foods may indicate that motivational or affective eating pathways remain active despite pharmacological enhancement of satiety (94).

This phenotype-informed approach may help avoid oversimplified interpretations. A patient with minimal weight loss should not automatically be considered non-adherent, and a patient reporting no clear “taste change” may still experience reduced appetite, altered meal size, or reduced cue-driven wanting. Conversely, persistent appetite, continued craving, or weight regain may indicate that psychological, behavioural, environmental, or treatment-exposure factors are overriding satiety-related effects, especially because weight regain can occur after treatment withdrawal or interruption (95). Distinguishing sensory, liking, wanting, and homeostatic response patterns may therefore improve clinical communication, nutritional counselling, and individualised treatment planning, in line with obesity pharmacotherapy guidance emphasising the combination of medication with lifestyle and behavioural management (96).

## 8 Research gaps and future directions

Current research on how GLP-1RAs affect eating experience is still quite fragmented, and the findings are not always easy to compare. Studies differ in the endpoints they use, the length of follow-up, and the way the data are analysed, which makes interpretation more difficult. In addition, most studies only focus on one level, such as sensory change, liking, or wanting, or only assess patients at one time point. As a result, there is still limited understanding of how these different levels may interact over time. Although existing studies suggest that GLP-1RAs can influence eating experience, the underlying mechanisms, time course, and individual differences are still not fully clear. At this point, the key challenge is no longer whether GLP-1RAs can alter eating experience, but how to define these effects more precisely, track their trajectory over time, and explain why they differ across patients.

### 8.1 Endpoint hierarchy and measurement standardisation

Current studies show substantial heterogeneity in endpoint selection (9, 97). Some focus on sensory measures such as taste sensitivity and taste-quality identification, others on subjective hedonic evaluation, and others on food craving or motivational drive. However, these endpoints correspond to different functional levels and do not share the same neural mechanisms or behavioural implications.

Without explicit distinction between these levels, grouping such outcomes together under the broad label of “taste change” may generate interpretive confusion. For example, the absence of psychophysical change does not exclude alteration in liking or wanting; conversely, reduced craving does not necessarily imply impairment of sensory input. In addition, measurement tools differ in sensitivity, reproducibility, and control of state-related confounding, further complicating comparison across studies.

A priority for future research is to define endpoint function explicitly at study design and to measure sensory, liking, and wanting in parallel whenever feasible.

### 8.2 Methodological limitations of human fMRI studies

In addition to endpoint heterogeneity, methodological limitations also constrain the interpretation of human functional magnetic resonance imaging (fMRI) evidence in this context. Although fMRI studies provide valuable non-invasive evidence that GLP-1RA treatment may alter neural responses to food-related cues, fMRI primarily measures blood-oxygen-level-dependent signals as an indirect index of neural activity, and therefore cannot determine whether GLP-1RAs act directly on specific central GLP-1R-expressing neuronal populations or indirectly through changes in peripheral physiology, interoceptive state, gastric emptying, or nausea-related signalling (98). Moreover, the temporal resolution of fMRI is limited by the haemodynamic response and is substantially poorer than the millisecond-scale neural resolution achievable in animal electrophysiological, optogenetic, or chemogenetic studies. This makes it difficult for human fMRI to resolve the rapid temporal sequence from food-cue perception to anticipatory motivation, hedonic appraisal, and post-ingestive feedback.

In addition, most food-cue fMRI paradigms rely on visual food images or anticipatory reward tasks, which may not fully capture the multisensory and consummatory processes involved in real eating behaviour. Importantly, “food reward” is not a unitary construct; hedonic “liking,” motivational “wanting,” incentive salience, and cue-induced craving may involve partly dissociable neural mechanisms (99, 100). Therefore, altered activation in reward-related regions should not be directly equated with changes in taste perception, hedonic experience, or food preference. Future studies should combine standardised food-cue paradigms with explicit behavioural measures of liking and wanting, assessment of interoceptive and nausea-related responses, and longitudinal or pharmacological designs to better clarify the causal contribution of central GLP-1 signalling to human taste, appetite, and reward processing.

### 8.3 Inter-individual variability, non-responders, atypical responses, and endpoint-dependent effects

Although GLP-1RAs show broadly consistent weight-loss efficacy at the population level, their effects on eating experience appear to vary substantially between individuals (71, 72). Some patients report marked reductions in food interest or changes in dietary preference, whereas others show little change. Importantly, this heterogeneity is not limited to the magnitude of weight loss, but may also include minimal treatment response, persistent appetite, weight regain, and, in rare cases, paradoxical increases in appetite and food intake.

For example, semaglutide 2.4 mg produces clinically meaningful mean weight loss in phase 3 trials, yet a minority of treated participants do not achieve conventional response thresholds (72). Similarly, early-response analyses with liraglutide 3.0 mg showed that early non-responders achieved substantially smaller long-term weight loss (93). Weight regain after semaglutide withdrawal in the STEP 1 extension further suggests that treatment-related changes in appetite and weight regulation may not persist uniformly after discontinuation (95). Rare case-level evidence of paradoxical appetite increase and progressive weight gain during semaglutide therapy also suggests that behavioural and psychological factors may counteract expected anorexigenic effects in selected individuals (101).

The available literature is also not uniformly consistent with a simple model in which GLP-1RAs consistently reduce food reward, hedonic liking, or motivational drive. Longer-term liraglutide fMRI data did not show consistent reductions in food-cue-related brain activation in the primary analysis, and adjustment for body weight or BMI revealed increased right orbitofrontal cortex activation in response to food cues (102). Similarly, an MRI-compatible gustometer study found liraglutide-induced weight loss without clear self-reported or neural evidence of reduced food-related liking during consumption (103). Preclinical data also suggest context dependence, as chronic semaglutide reduced chow intake and body weight in rats but increased intake of low- to mid-range sucrose solutions under specific testing conditions (104). These findings suggest that GLP-1RA-related changes in eating experience should be interpreted as heterogeneous, endpoint-dependent, and context-dependent rather than as a uniform suppression of reward or motivation.

Most current studies are based on average group effects and do not provide a systematic classification of response patterns. For example, it remains unclear which individuals are more likely to show an early state-dependent response and which are more likely to exhibit sustained reduction in motivational drive. It also remains unclear which patients may show limited response, persistent appetite, weight regain, or paradoxical appetite responses despite treatment.

Potential contributors may include baseline reward sensitivity, emotional eating tendency, metabolic status, diabetes status, medication adherence, gastrointestinal tolerability, prior dietary patterns, and broader psychological context, but these variables have not yet been systematically integrated. A useful next step will be to integrate behavioural, physiological, and neuroimaging data so that clinically meaningful response subtypes and their predictors can be identified more reliably, with potential relevance for more individualised treatment planning. To provide a concise overview of these heterogeneous findings, representative studies reporting divergent, atypical, or endpoint-dependent responses to GLP-1RA treatment are summarised in Table 2.

**Table 2:** Divergent responses to GLP-1RA treatment.

Study/model	Treatment	Divergent finding	Interpretation
STEP 1 trial (72)	Semaglutide 2.4 mg	Some participants did not reach response thresholds.	Individual responses vary.
Liraglutide early-response analysis (93)	Liraglutide 3.0 mg	Early non-responders had weaker long-term outcomes.	Early response may identify subtypes.
STEP withdrawal studies (95)	Semaglutide withdrawal	Weight regain occurred after withdrawal.	Effects may require continued exposure.
Liraglutide fMRI study (102)	Liraglutide	Food-cue brain activation was not uniformly reduced.	Reward effects are endpoint-dependent.
Gustometer RCT (103)	Liraglutide	Weight loss occurred without clear reduction in liking.	Weight loss and liking may dissociate.
Rat sucrose-intake study (104)	Semaglutide	Sucrose intake increased under some conditions.	Food-choice effects may be context-dependent.
Case report (101)	Semaglutide	Paradoxical appetite increase and weight gain occurred.	Behavioural factors may modify response.

### 8.4 Psychological and behavioural moderators of eating-experience response

Psychological and behavioural factors may be important moderators of eating-experience responses during GLP-1RA treatment and may help explain why changes in appetite, food reward, and food motivation are not uniform across individuals. Emotional eating, binge-eating tendencies, psychiatric comorbidity, learned eating behaviour, reward sensitivity, and baseline dietary patterns may all shape how patients perceive and respond to GLP-1RA-induced changes in satiety, nausea, food preference, and hedonic evaluation.

Existing evidence suggests that GLP-1RAs may influence binge-eating symptoms and eating-disorder-related psychopathology, but the effects are heterogeneous and depend strongly on study design, behavioural context, and patient phenotype. In an exploratory trial of liraglutide combined with intensive behavioural therapy, short-term improvements were observed in dietary disinhibition, eating-disorder psychopathology, and shape concern; however, group differences were no longer significant at 52 weeks, and total food cravings did not differ between groups (94). Similarly, in a pilot randomised trial for binge-eating disorder, liraglutide produced greater weight loss than placebo, but reductions in objective binge episodes and binge remission did not differ significantly between groups, suggesting that weight loss and binge-eating improvement may not always change in parallel (105). Conversely, an open-label retrospective cohort reported greater reductions in Binge Eating Scale scores among patients receiving semaglutide, and recent reviews have suggested potential benefits of GLP-1RAs for binge-eating behaviour, although large-scale, blinded, placebo-controlled trials remain limited (106, 107). At the same time, caution is warranted in patients with current or previous eating disorders or psychiatric comorbidity, because GLP-1RAs may theoretically improve, maintain, or worsen eating-disorder symptoms depending on the individual context (108).

Therefore, psychological and behavioural variables should not be treated merely as background characteristics, but as potential moderators of treatment response. Future studies should prospectively assess emotional eating, binge-eating symptoms, psychiatric comorbidity, reward sensitivity, learned dietary patterns, and baseline food preferences alongside taste perception, satiety, nausea, liking, wanting, and weight outcomes. Such an approach may help identify clinically meaningful psychological or behavioural phenotypes that predict different eating-experience responses to GLP-1RA therapy.

### 8.5 Extension to dual, triple-agonist and amylin-based co-agonist therapies

The emergence of dual and multi-agonist therapies, such as the GLP-1 and glucose-dependent insulinotropic polypeptide (GIP) receptor agonist tirzepatide, raises important questions regarding the generalisability of the proposed framework. While these agents demonstrate greater weight loss efficacy, their effects on eating experience and food reward remain incompletely characterised.

GIP signalling has been implicated in both central and peripheral metabolic regulation, and may interact with reward pathways in ways that differ from or complement GLP-1 signalling. It is therefore plausible that dual agonists exert additive or distinct effects on hedonic evaluation and incentive motivation.

Beyond GLP-1/GIP dual agonism, triple-hormone receptor agonists such as retatrutide further expand this framework by engaging GIP, GLP-1, and glucagon receptors within a single molecule (109). Although retatrutide has shown substantial weight-loss efficacy in adults with obesity, the relative contributions of reduced appetite, altered food reward, enhanced energy expenditure, and glucagon-related metabolic effects remain incompletely understood (109). Importantly, whether triple agonists produce distinct effects on central reward circuits, including food-cue responses within VTA–NAc–OFC-related pathways, remains to be directly tested.

In parallel, amylin-based co-agonist strategies, exemplified by the long-acting amylin analogue cagrilintide and its combination with semaglutide, introduce an additional satiety-related signalling axis (110). Cagrilintide acts through amylin and calcitonin receptor systems, and recent evidence suggests that its body-weight-lowering effects are mediated, at least in part, through brain amylin receptor 1 and 3 signalling (111). Because amylin signalling is closely linked to meal termination, satiation, and brainstem–hypothalamic regulation of food intake, amylin–GLP-1 co-agonism may reshape eating experience through mechanisms that partly overlap with, but are not identical to, classical GLP-1R agonism. These emerging therapies therefore require direct assessment of taste perception, satiety, nausea, food preference, liking, and wanting rather than extrapolation from GLP-1RA data alone. Whether the sensory–liking–wanting dissociation proposed for GLP-1RAs is maintained, strengthened, or modified under dual, triple-agonist and amylin-based co-agonist therapy remains uncertain. Addressing this question will be important for both mechanistic refinement and more informed treatment selection in obesity management.

### 8.6 Limitations of the current evidence base

Several limitations of the current evidence base should be acknowledged. Much of the available human evidence is observational, secondary, or exploratory in nature, and many studies were not specifically designed to distinguish sensory function, hedonic liking, motivational wanting, and broader dietary behaviour. Existing studies also differ substantially in population, drug type, dose, treatment duration, follow-up window, endpoint definition, and control of gastrointestinal symptoms or other state-related confounders. In addition, patient-reported “taste change” and food-preference outcomes are often not measured using standardised instruments, making comparisons across studies difficult. Finally, although neuroimaging and behavioural studies provide important mechanistic clues, they generally do not establish causal relationships between GLP-1RA exposure, central reward-circuit modulation, altered liking or wanting, and long-term eating behaviour in humans. These limitations reinforce the need to interpret the proposed sensory–liking–wanting framework as a conceptual model and to test it prospectively in longitudinal, multimodal studies.

## 9 Conclusion

GLP-1RA-associated changes in eating experience should not be reduced to a uniform taste-abnormality model. Current evidence is more consistent with a multilevel process in which peripheral modulation within taste pathways, brainstem integration of gustatory and interoceptive signals, and central changes in food valuation interact to shape how food is experienced during treatment. Within this framework, many patient-reported “taste changes” are more plausibly interpreted as reflecting altered hedonic evaluation or reduced motivational salience rather than a stable primary disturbance of basic gustatory function.

A sensory-liking-wanting framework may therefore provide a more precise and biologically grounded way to interpret the heterogeneity of current findings. Importantly, reduced food intake during GLP-1RA therapy may arise from overlapping but non-equivalent mechanisms, including homeostatic suppression, interoceptive-state-dependent revaluation, and reward-related downregulation of food cue value. Distinguishing these processes is essential for interpreting both mechanistic studies and patient-reported outcomes.

Accordingly, the sensory–liking–wanting framework should be viewed as a conceptual and hypothesis-generating structure for interpreting GLP-1RA-associated eating-experience phenotypes, rather than as a validated causal model. Future research should prioritise longitudinal and multimodal designs that assess sensory function, hedonic response, motivational drive, and state-related symptoms in parallel. Such approaches will be necessary to determine when altered eating experience reflects transient adaptive effects, clinically meaningful nutritional consequences, persistent remodelling of food reward, or different combinations of these processes across individuals receiving GLP-1RA therapy.
